# Concentration of educational attainment and age-specific risk patterns of preventable avoidable mortality in Hungary, 2007–2023

**DOI:** 10.3389/fpubh.2026.1838674

**Published:** 2026-07-13

**Authors:** B. Burkali, A. Juhász, B. Oroszi, R. Ádány, Cs. Nagy

**Affiliations:** 1 Doctoral School of Health Sciences, University of Debrecen, Debrecen, Hungary; 2 Department of Quality Management in Healthcare and Infection Control, Petz Aladár Teaching Hospital of Gyor-Moson-Sopron County, Gyor, Hungary; 3 Center for Epidemiology and Surveillance, Semmelweis University, National Laboratory for Health Security, Budapest, Hungary; 4 Department of Public Health and Epidemiology, Faculty of Medicine, University of Debrecen, Debrecen, Hungary; 5 HUN-REN-UD Public Health Research Group, University of Debrecen, Debrecen, Hungary; 6 Department of Preventive Medicine and Public Health, Semmelweis University, Budapest, Hungary

**Keywords:** cumulative disadvantage, deprivation, educational attainment, life-course perspective, preventable avoidable mortality, social gradient

## Abstract

Educational attainment is one of the most consequential social determinants of health, fundamentally shaping mortality risks and the patterns of health inequalities. The international literature has extensively documented a close association between socioeconomic disadvantage and poor health—a relationship that is particularly pronounced in post-socialist countries, including Hungary. Hungarian studies likewise show that social deprivation particularly the education is not merely a background determinant but an independent health-shaping factor, producing clearly detectable differences in avoidable mortality even at the settlement-level. The relationship between mortality risk and educational attainment was modelled using negative binomial regression with a log-link function, applying log-transformed population counts as an offset variable. Associations were examined by age, sex, and settlement type, allowing for age-specific analysis of the impact of education on preventable avoidable mortality (PAM) risk. Our results confirm that PAM is most strongly associated with the concentration of low-educated populations, along which a pronounced social gradient emerges. In areas with the highest concentration of low-educated populations, the mortality risk was nearly 40% higher compared to settlements in the lowest quintile of low-educated concentration while a high concentration of tertiary education was associated with a reduction in mortality risk of similar magnitude. The gradient was consistent across all educational levels and was strongest and most linear among the least educated. Age-specific models revealed that the effect of education peaked in the 35–54 age group and gradually weakened in older populations, consistent with the theories of cumulative disadvantage and the survivor effect. While tertiary education was strongly associated with lower mortality risk and low education was associated with substantially higher mortality risk, the association among vocationally trained groups was weak and heterogeneous. Overall, preventable mortality proved highly sensitive to educational inequalities, underscoring that investment in education—particularly the training of disadvantaged groups—remains one of the most effective strategies for improving population health and reducing social disparities.

## 1 Introduction

It is well documented in the international literature that socioeconomic disadvantage and poor health are closely interrelated. According to Marmot’s seminal work (1), an individual’s social position largely determines both the health risks they are exposed to and their opportunities to mitigate these risks. This effect begins in childhood and can persist throughout the life course. Findings from a major European comparative study further confirm that social inequalities in health are significant in most countries, but post-socialist societies—including Hungary—are in a particularly unfavourable situation in this regard (2–4). This observation is further supported by a large retrospective cohort study conducted in three Eastern European countries, which found that, following the socio-political changes of the early 1990s, inequalities in mortality according to educational level continued to grow in Russia, Belarus and Hungary (5).

It is also well established that within Hungary there are disadvantaged regions—particularly in the north-eastern and eastern parts of the country—where multiple unfavourable socioeconomic factors are concentrated, showing a strong correlation with premature mortality. This association is especially pronounced in the case of cardiovascular diseases and malignant neoplasms (6–9), but it has also been observed for premature deaths related to COVID-19 (10, 11). Social deprivation, therefore, should not be viewed merely as a “background factor” but as an independent determinant of health.

The impact of social deprivation on health outcomes is not only evident in worse overall health status or a reduced life expectancy; it is most importantly seen in avoidable mortality, one of the most significant indicators of health inequalities. In this regard, a series of international studies over the past decades (12, 13), as well as several Hungarian investigations (14), have confirmed that avoidable mortality is strongly associated with socioeconomic status. Comprehensive international analyses (15, 16) focusing on healthcare-amenable mortality have revealed substantial systemic differences between countries in terms of social structure and health policy performance. In countries characterized by higher social cohesion and a more integrated healthcare system, the level of avoidable mortality was significantly lower (17).

Socioeconomic composite indices are useful tools for illustrating social cohesion and measuring inequalities. Among the factors included in such multidimensional deprivation indices, educational attainment carries particular weight, exerting both direct and indirect effects on health outcomes through multiple pathways. Numerous studies have demonstrated that educational level is closely associated with a wide range of health outcomes, including the prevalence of chronic diseases, life expectancy, self-rated health, and interaction with healthcare services (18, 19). According to Cutler and Lleras-Muney’s seminal study (20) education influences nearly all known determinants of health, some of which—such as smoking habits, physical activity, and nutrition—directly affect mortality risk through preventive behaviour. Hungarian research has also provided compelling evidence of the decisive role of educational attainment in shaping individual life prospects. Demographic analysis (21) showed that by 2011, the gap in life expectancy between individuals without formal education and those with a university degree exceeded 15 years for men and 10 years for women. In this context, formal education simply refers to education acquired within the structured school system; therefore, completion of primary school is also considered formal education. Consistent with this pattern, a recent meta-analysis estimated that an adult with 12 years of schooling has, on average, a 24.5% lower risk of mortality than an adult with no formal education, corresponding to an average reduction of 1.9% in mortality risk for each additional year of schooling (18).

Although the impact of education on health is substantial and multifaceted, it is not constant across the life course. Research indicates that its effect is strongest among young and middle-aged populations, while it gradually diminishes in older age, as its influence on health outcomes weakens. Furthermore, recent European evidence indicates that educational inequalities in mortality have not only persisted over successive birth cohorts but have also widened in most of the countries included in the study (22). In younger age groups, education primarily affects health through its influence on health behaviours (23). Among middle-aged individuals, in addition to shaping health behaviour, educational attainment also affects stress levels, housing conditions, and access to healthcare services through its mediation of labour market position and social status.

This latter aspect is a particularly important determinant of healthcare-amenable avoidable mortality (17). Although previous Hungarian studies have examined the relationship between avoidable mortality and socioeconomic status (24), they did not address inequalities in avoidable mortality that are preventable through primary prevention, nor their relationship with deprivation. Yet the alarmingly high level of primary-prevention-amenable avoidable mortality in the Hungarian population in a European context (25, 26) along with the well-documented spatial inequalities associated with deprivation within the country, make it essential to analyse the interplay between these two dimensions. Furthermore, as educational level—a key factor influencing primary prevention—and its impact on health vary across age groups, it is necessary to investigate how this effect manifests at different stages of life.

Considering these points, this ecological study aims, first, to identify the relationship between preventable avoidable mortality (PAM) and educational attainment at the settlement-level, and second, to provide empirical evidence for the association between age-specific patterns of preventable mortality and local educational structures.

This study is the first nationwide ecological analysis in Hungary to examine the association between settlement-level educational attainment and PAM across different stages of the adult life course. Beyond demonstrating a strong relationship between educational inequalities and PAM, it identifies middle adulthood—particularly the 45–54-year age group—as the period during which educational attainment exerts its greatest influence on PAM. By revealing the age-specific dynamics of educational inequalities in preventable mortality, the study extends the existing literature on the life-course dimension of social inequalities in health and provides an empirical basis for more precisely targeted public health interventions.

The remainder of the paper is organized as follows. Section 2 describes the data sources and methodological approach. Section 3 presents the results. Section 4 discusses the findings in the context of previous research and relevant theoretical frameworks. Section 5 outlines the public health and policy implications of the findings.

## 2 Data and methods

### 2.1 Data

The analysis was based on comprehensive, settlement-level data on mortality from causes amenable to primary prevention in Hungary for the period 2007–2023. The group of causes of death considered preventable through primary prevention was defined according to the joint OECD/Eurostat list of avoidable causes of death, which is applicable to the population aged 0–74 years (46). This category includes a broad range of causes of death, including infectious diseases, selected malignant neoplasms, cardiovascular and respiratory diseases, alcohol- and drug-related conditions, and external causes such as injuries and transport accidents. For the purposes of the present analysis, however, the study population was restricted to adults aged 25–74 years. Individuals younger than 25 years were excluded due to the low number of preventable mortality events and because educational attainment is not yet fully established in these age groups, particularly with respect to tertiary education.

Mortality data (by settlement, year, sex, and 10-year age group) were obtained from the Hungarian Central Statistical Office, while population data were provided by the Deputy State Secretariat for the Management of Registers of the Ministry of Interior. Data on the settlement-level educational structure were derived from the results of the 2022 Population Census, also provided by the HCSO.

## 3 Methods

### 3.1 Mapping

The education level maps are based on the 2022 Census Database of the Hungarian Central Statistical Office (Népszámlálási adatbázis—(27)). These visualisations illustrate the broader socio-geographical context of our findings. Their aim is to convey the characteristic features of the spatial distribution of educational attainment, which fundamentally shape the interpretive framework of the quintile-based models. Each map depicts, at the settlement-level, the density of educational categories according to their quintile classification, thereby revealing the regional concentrations and spatial patterns of the lower- and higher-educated populations in Hungary.

### 3.2 Association between the risk of PAM and educational attainment

In this part of the analysis, mortality risk was examined according to quintiles of educational concentration, complemented by variables for sex, age (five 10-year age groups: 25–34, 35–44, 45–54, and 55–64, as well as 65–74 years), and settlement type (capital, city with county rights, small town, village). Educational concentration does not describe the simple percentage distribution of educational attainment, but rather operationalizes its spatial-social concentration, that is, the extent to which a given level of education is represented within the social structure of a settlement. We calculated the quintiles by ranking all settlements according to the proportion of the population belonging to a given educational attainment category and then dividing this distribution into five equal groups (quintiles). Thus, each quintile represents 20% of all settlements, ranging from the lowest to the highest concentration of the given educational attainment category.

The following indicators were used to describe the educational structure of the population based on the methodological documentation of the Hungarian Central Statistical Office (28):

- the share of the population of corresponding age with at most 8 years of primary schooling (“low-educated”),
- the share of the population of corresponding age who have completed a vocational qualification (“vocationally trained”),
- the share of the population of corresponding age with a completed secondary school certificate (“upper secondary educated”),
- the share of the population of corresponding age holding a tertiary degree (“tertiary educated”).

For each level of educational attainment, the quintile categories were defined consistently: Q1 represented the lowest and Q5 the highest concentration of the given educational attainment category within the settlement, allowing for direct comparison of trends across the four separate models.

The analysis employed a Generalized Linear Model (GLM) with a negative binomial distribution and a log-link function. Generalized linear models are particularly suitable for non-normally distributed outcomes because they allow the specification of an appropriate probability distribution and link function according to the characteristics of the response variable (29). A negative binomial specification was chosen because mortality counts constitute overdispersed count data, for which standard linear regression is not appropriate and Poisson regression may underestimate variability by assuming equality of the mean and variance (30, 31). The negative binomial model accommodates overdispersion through an additional dispersion parameter while converging to the Poisson model when overdispersion is absent (31). The log-link function allows the estimation of relative mortality risks through exponentiated coefficients [Exp(B)]. To account for potential biases arising from differences in the age-specific population distribution across settlements, a log-transformed population offset variable (logpop2) was included in the models.

The dependent variable was the aggregated indicator of mortality amenable to primary prevention. The explanatory variables included the quintiles of educational attainment, sex, and settlement type. This approach allowed for the exploration of how the relationship between education and mortality is modified by the socio-spatial context, and how gender differences manifest across different educational and settlement environments.

Results are presented as Incidence Rate Ratios [IRR = Exp(B)], accompanied by 95% confidence intervals and significance levels.

**Figure 1:**
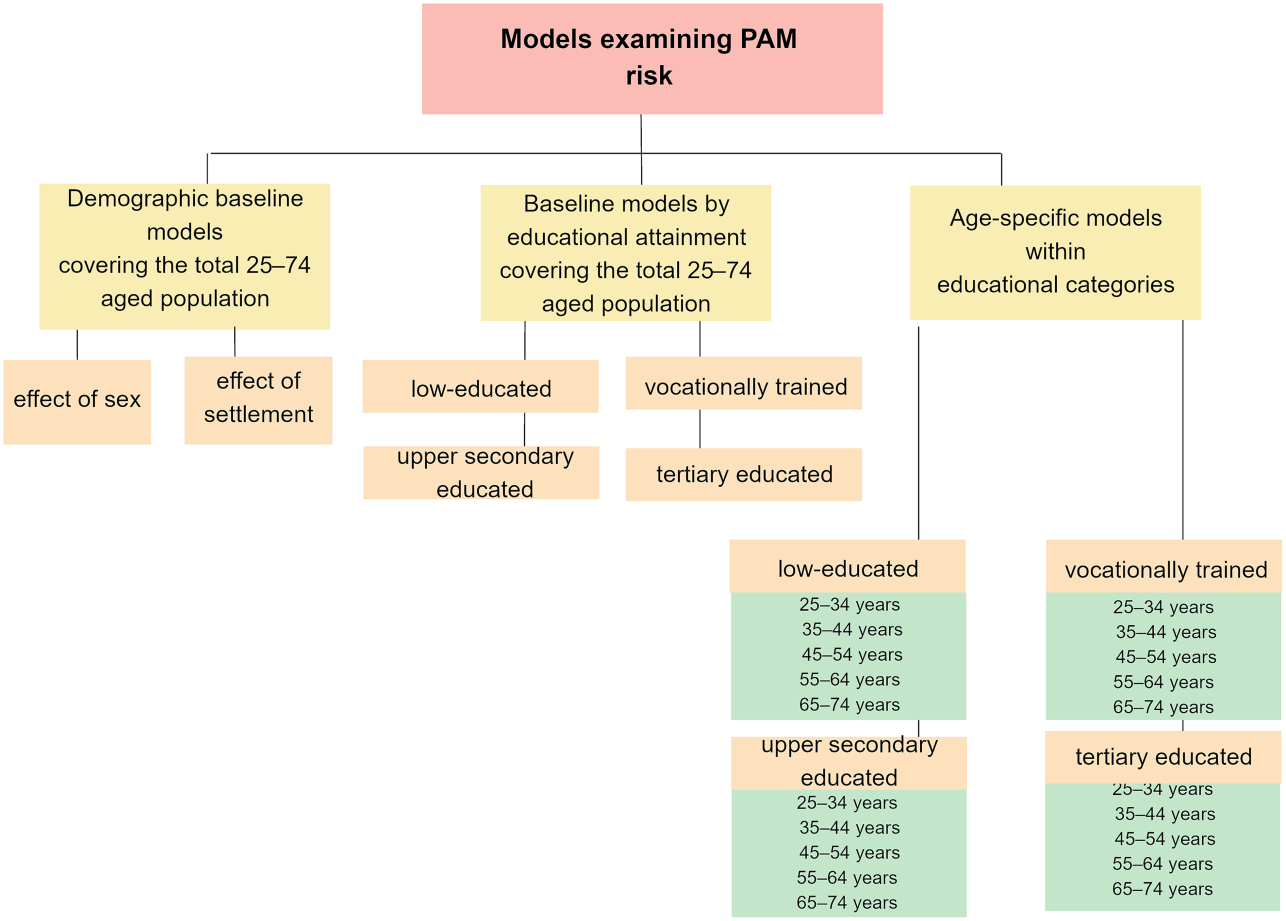
Hierarchical modelling framework for examining the association between educational concentration and PAM risk.

The study was based on the following models (Figure 1):

- *Demographic baseline models* (covering the total population aged 25–74 years)
  Examining the effects of sex and settlement type.
- *Baseline models by educational* attainment (for the total population aged 25–74 years)
  Four separate models were built for the following educational categories, in which the predictor variable was the quintile of the respective educational level:
  low-educated,
  vocationally trained,
  upper secondary educated,
  tertiary educated.
- *Age-specific models within educational categories*
  Within each of the four educational categories, additional submodels were constructed by age, using five 10-year age groups:
  25–34 years,
  35–44 years,
  45–54 years,
  55–64 years,
  65–74 years.

The age-specific analyses were based on 8,371 PAM deaths in the 25–34 age group, 23,036 PAM deaths in the 35–44 age group, 78,068 PAM deaths in the 45–54 age group, 182,212 PAM deaths in the 55–64 age group, and 246,829 PAM deaths in the 65–74 age group, demonstrating that the age-specific analyses were based on a substantial number of events in each age group.

Thus, each educational level included five age-specific models, resulting in a total of 20 submodels. The purpose of these was to explore how the effect of educational concentration on PAM risk changes with advancing age, and to identify the life stage at which the structural disadvantage associated with educational attainment exerts its strongest impact.

To assess the robustness of the models, a sensitivity analysis was performed in which the original age-specific models were re-estimated after adjustment for sex and settlement type. The results showed that the estimates obtained from the adjusted models were consistent with those of the original models: effect sizes changed only negligibly, while the direction of the associations and the overall patterns remained unchanged across all age groups.

The goodness of fit of the negative binomial regression models fitted for the different educational attainment groups was assessed using the Omnibus Likelihood Ratio Chi-Square (LR*χ*^2^) statistic and the Akaike Information Criterion (AIC) values.

Within each educational attainment group, the goodness of fit of the age-specific regression models and the strength of the association between social concentration and mortality were compared based on multiple criteria. The following indicators were used for the evaluation:

1. *Magnitude of risk change*: Based on the incidence rate ratio [Exp(B)] values derived from the regression models, the extent to which mortality risk varied within each educational category compared with the fifth quintile we examined.
2. *Overall model fit*: The general goodness of fit of each model was assessed using the Omnibus LR*χ*^2^ statistic. A higher LR*χ*^2^ value indicates a stronger model fit and a more pronounced association between educational concentration and mortality.

The accompanying table, based on the combined assessment of these two indicators, enabled the identification of the age groups in which the effect of social concentration on mortality risk avoidable to primary prevention was the strongest.

IBM SPSS Statistics version 29.0 was used for all analyses (32).

## 4 Results

### 4.1 Association between the risk of PAM and sociodemographic factors (sex, settlement type, and educational attainment)

Among the demographic factors, sex proved to be the strongest determinant: the mortality risk among men was more than 2.5 times that among women [Exp(B) = 2.564; 95% CI: 2.529–2.600; *p* < 0.001].

Settlement type also had a significant effect on mortality, even after controlling for population size. The mortality risk among residents of the capital was 27% lower than among those living in villages [reference group; Exp(B) = 0.731; 95% CI: 0.699–0.764; *p* < 0.001]. In cities with county rights, the risk was reduced by 12% [Exp(B) = 0.878; 95% CI: 0.835–0.922; *p* < 0.001], whereas in smaller towns only a modest 5% reduction was observed [Exp(B) = 0.954; 95% CI: 0.939–0.970; *p* < 0.001].

Based on population-level mortality data, we also examined the relationship between the territorial concentration of educational attainment and mortality at settlement-level.

The adult population of Hungary was stratified into clearly distinguishable social layers using quintiles derived from educational attainment. The proportions of those with at most primary education and those with tertiary education were almost identical at the national level, each representing about 22–23% of the population. A similar share was observed among individuals with vocational qualifications (21.1%), while the largest group—more than one-third of adults—consisted of those with upper secondary education [Census Database–(27, 47)].

**Figure 2:**
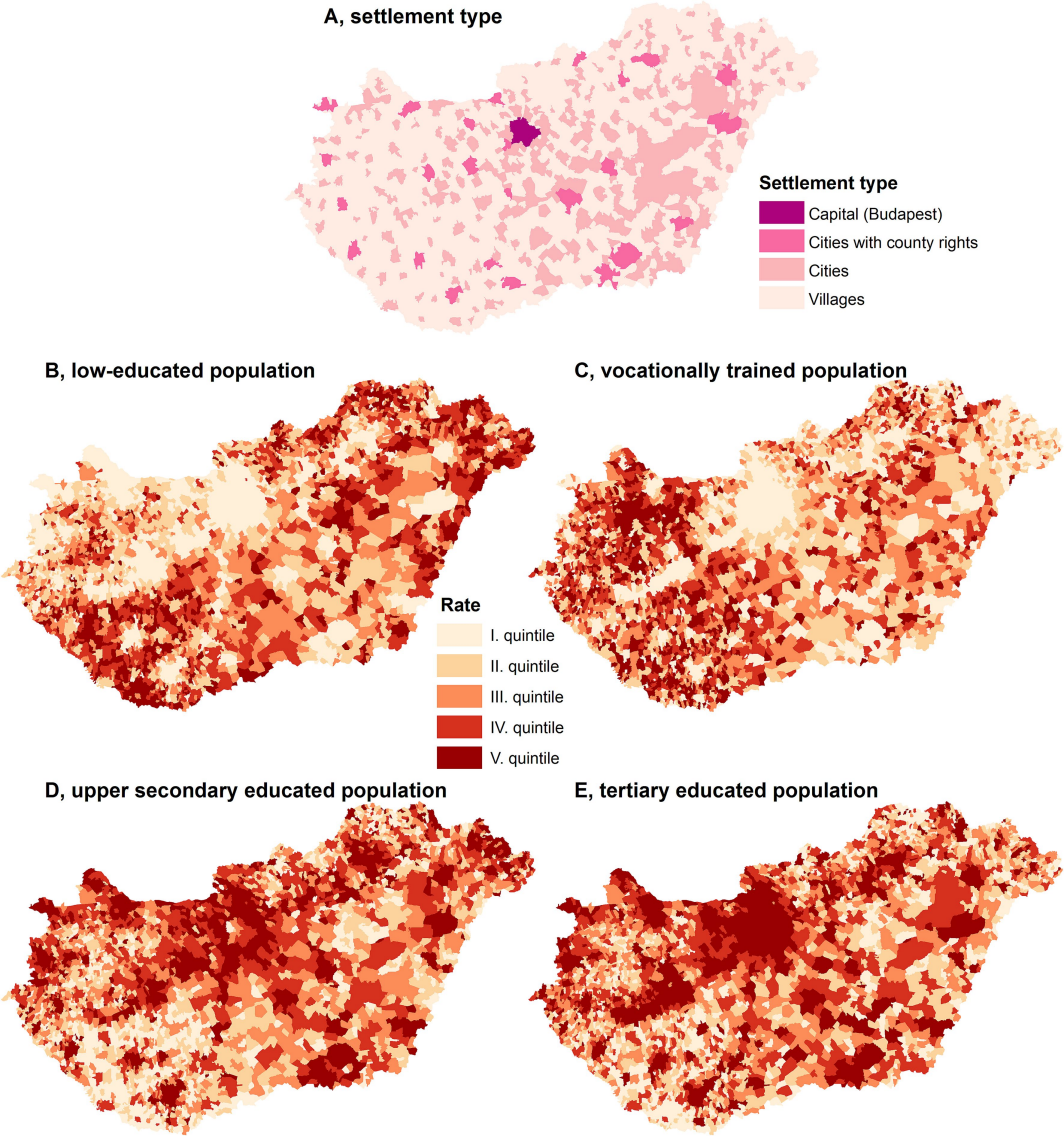
Spatial distribution of settlements **(A)** and the adult population in Hungary by educational attainment (**B**: low-educated, **C**: vocationally trained, **D**: upper secondary educated, **E**: tertiary educated population), 2022.

This quasi “four-tier” social structure, however, exhibits strong spatial differentiation, as clearly illustrated by the map-based analyses. The maps highlight the concentration patterns, showing the predominance of lower educational levels in deprived regions and the higher educational profiles characteristic of the capital and city with county rights (Figure 2A).

**Figure 3:**
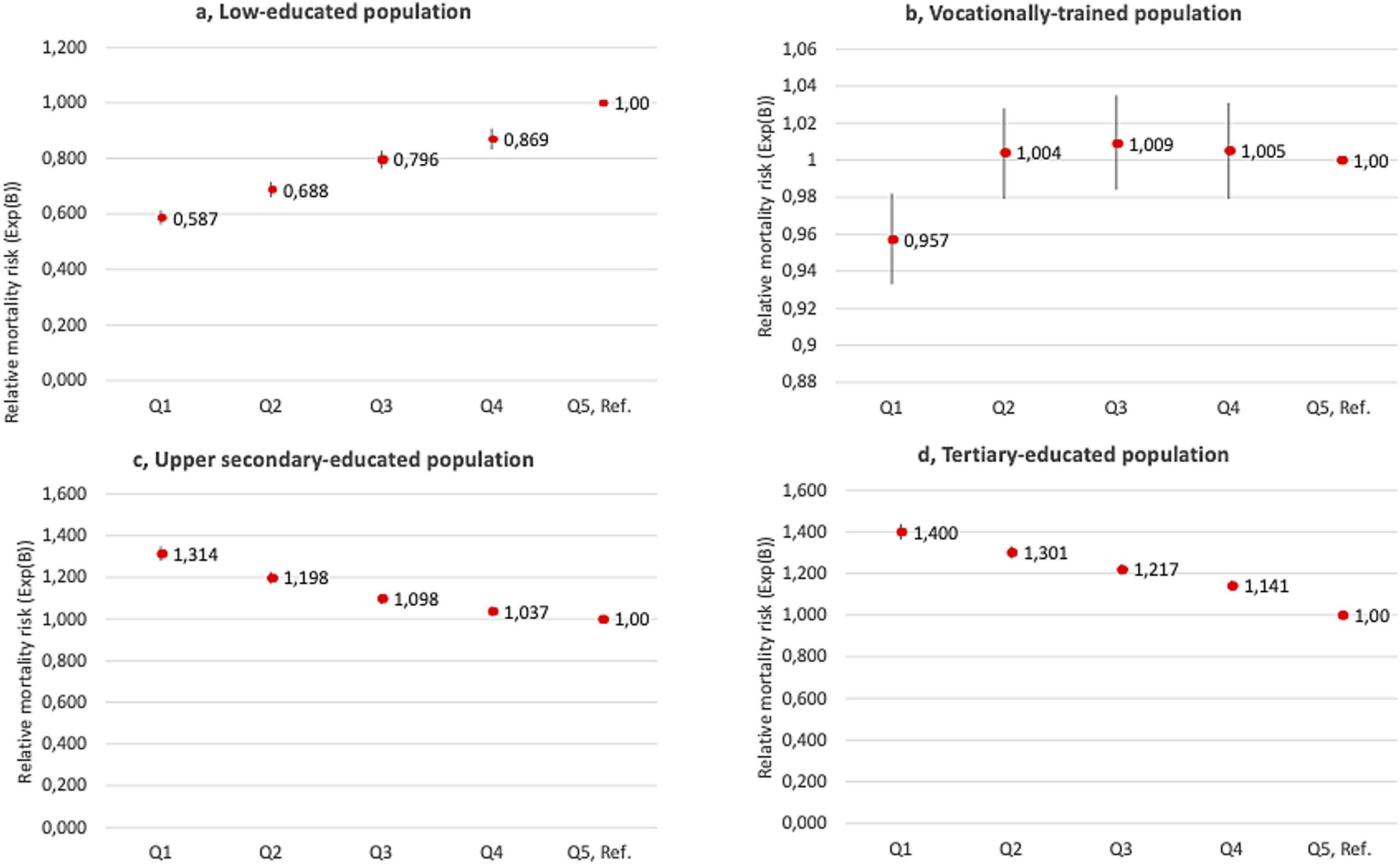
Association of educational attainment quintiles on the relative risk of PAM [Exp(B), 95% CI; **a**: low-educated, **b**: vocationally trained, **c**: upper secondary educated, **d**: tertiary educated population].

In settlements belonging to the first quintile, the risk of mortality amenable to primary prevention was nearly 40% lower [Exp(B) = 0.587; 95% CI: 0.563–0.613; *p* < 0.001] compared with settlements in the fifth quintile (reference group) with the highest share of low-educated population. Even in areas belonging to the fourth quintile, a significant reduction in risk was observed [Exp(B) = 0.869; 95% CI: 0.833–0.907; *p* < 0.001; Figure 3a].

In the case of the vocationally trained population, a significant difference was observed only in the first quintile [Exp(B) = 0.957; 95% CI: 0.933–0.982; *p* < 0.001], whereas the relative risks in the second to fourth quintiles did not differ significantly from the reference category (Figure 3b).

Among the population with upper secondary education, a statistically significant association was also found between mortality risk and the degree of educational concentration. The excess risk in settlements belonging to the lowest quintile of upper secondary attainment exceeded 30% [Exp(B) = 1.314; 95% CI: 1.279–1.349; *p* < 0.001]. As the proportion of upper secondary educated increased, the magnitude of excess risk decreased proportionally (Figure 3c).

Mortality risk showed a significant and gradual increase with decreasing concentration of the tertiary educated population. According to the regression model, compared with areas with the highest proportion of tertiary educated residents, mortality risk was already 14.1% higher in the fourth quintile [Exp(B) = 1.141; 95% CI: 1.116–1.167; *p* < 0.001]. In regions with the lowest concentration of tertiary educated population, the risk of mortality was 40.0% higher [Exp(B) = 1.400; 95% CI: 1.363–1.437; *p* < 0.001]. The trend was linear, and the differences between all quintiles were statistically significant. The increase followed an almost uniform step of 6–9% per quintile (Figure 3d).

Although a statistically significant predictive effect was identified in all models, the regression models separated by educational level captured the variance in mortality risk to different extents, as indicated by the model fit statistics.

The model based on the low-educated population showed the best model fit (Omnibus LR*χ*^2^ = 1,344.827; df = 4; *p* < 0.001), while its AIC value (477,473.001) was the lowest among all models (Table 1).

**Table 1:** Model fit indicators of the negative binomial regression models examining the association between educational concentration and PAM (Omnibus test, df, *p*, AIC).

Age group	Omnibus LR*χ*^2^	df	*p*	AIC
Low-educated population	1,344.827	4	<0.001	477,473.001
Vocationally trained population	26.917	4	<0.001	478,790.912
Upper secondary educated population	561.824	4	<0.001	478,256.004
Tertiary educated population	798.346	4	<0.001	478,019.482

The second strongest model described the mortality pattern of the tertiary educated population (Omnibus LR*χ*^2^ = 798.346; df = 4; *p* < 0.001), although its AIC value (478,019.482) was higher. Among the upper secondary educated population, the model showed a more moderate fit (LR*χ*^2^ = 561.824; df = 4; *p* < 0.001, AIC = 478,256.004). The model for the vocationally trained group showed the weakest fit (LR*χ*^2^ = 26.917; df = 4; *p* < 0.001; AIC = 478,790.912), indicating only a minimal effect and poorer fit (Table 1).

### 4.2 Relationship between PAM risk across educational groups and age categories

#### 4.2.1 Low-educated population

Among the low-educated population aged 25–34, the association between social concentration and mortality risk was already detectable, although the pattern was not yet fully structured. In areas with the lowest concentration of low-educated residents, mortality risk was 56.1% lower compared with the fifth quintile, representing the highest concentration [Exp(B) = 0.439; 95% CI: 0.363–0.530; *p* < 0.001; Table 2].

**Table 2:** Age-specific associations between the settlement-level concentration of the low-educated population and PAM risk [Exp(B), 95% CI].

Age group	Low-educated population	*p*-value	Exp(B)	95% confidence interval for Exp(B)	
				Lower	Upper
25–34 years old	Q1	<0.001	0.439	0.363	0.530
	Q2	<0.001	0.586	0.488	0.705
	Q3	<0.001	0.686	0.570	0.826
	Q4	0.007	0.764	0.628	0.930
	Q5, Ref.	–	1	–	–
35–44 years old	Q1	<0.001	0.434	0.377	0.500
	Q2	<0.001	0.574	0.501	0.657
	Q3	<0.001	0.726	0.634	0.832
	Q4	0.023	0.848	0.735	0.978
	Q5, Ref.	–	1	–	–
45–54 years old	Q1	<0.001	0.514	0.464	0.570
	Q2	<0.001	0.649	0.588	0.715
	Q3	<0.001	0.784	0.712	0.864
	Q4	0.010	0.876	0.792	0.968
	Q5, Ref.	–	1	–	–
55–64 years old	Q1	<0.001	0.529	0.485	0.577
	Q2	<0.001	0.626	0.577	0.679
	Q3	<0.001	0.721	0.665	0.782
	Q4	<0.001	0.809	0.743	0.880
	Q5, Ref.	–	1	–	–
65–74 years old	Q1	<0.001	0.639	0.587	0.695
	Q2	<0.001	0.714	0.660	0.774
	Q3	<0.001	0.813	0.752	0.880
	Q4	0.002	0.880	0.811	0.956
	Q5, Ref.	–	1	–	–

In the 35–44 age group, the inverse association between PAM risk and social concentration became more clearly defined, showing a pattern remarkably similar to that observed among the 25–34-year-olds: in both age groups, a steady, linear social gradient emerged, organized along the spatial concentration of the low-educated population. Mortality risk in the first quintile was 56.6% lower than in the fifth quintile [Exp(B) = 0.434; 95% CI: 0.377–0.500; *p* < 0.001], while the effect remained statistically significant across all intermediate quintiles.

In the 45–54 age group, the relationship persisted and showed a more coherent gradient structure across quintiles. According to the model, mortality risk was significantly lower in all quintiles compared with the fifth quintile. The largest difference remained in the first quintile [Exp(B) = 0.514; 95% CI: 0.464–0.570; *p* < 0.001], but a clear reduction in risk was also observed in the second to fourth quintiles.

Among those aged 55–64, the social gradient remained clearly visible, and its structure and trend were almost identical to the pattern observed in the 45–54 age group. In the first quintile, the PAM risk was 47.1% lower than in the fifth quintile [Exp(B) = 0.529; 95% CI: 0.485–0.577; *p* < 0.001], while the reduction in risk was consistent across all quintile.

In the 65–74 age group, the relationship between social concentration and mortality risk remained statistically significant; however, the intensity of the effect declined compared with younger age groups. Mortality risk in areas with the lowest concentration remained 36.1% lower than in those with the highest concentration [Exp(B) = 0.639; 95% CI: 0.587–0.695; *p* < 0.001], while the gradient became less steep, and the differences in risk between quintiles gradually diminished.

#### 4.2.2 Vocationally trained population

Among the vocationally trained population aged 25–34, the relationship between mortality risk and the social concentration of vocational education was statistically significant but did not show a gradual, quintile-based progressive pattern. Mortality risk was significantly lower in areas with the lowest proportion of vocationally trained residents [Exp(B) = 0.804; 95% CI: 0.713–0.907; *p* < 0.001] compared with the reference group—the regions with the highest vocational concentration—while no significant differences were observed in the second to fourth quintiles (*p* > 0.05; Table 3).

**Table 3:** Age-specific associations between the settlement-level concentration of the vocationally trained population and PAM risk [Exp(B), 95% CI].

Age group	Vocationally trained population	*p*-value	Exp(B)	95% confidence interval for Exp(B)	
				Lower	Upper
25–34 years old	Q1	<0.001	0.804	0.713	0.907
	Q2	0.301	0.938	0.830	1.059
	Q3	0.449	0.953	0.842	1.079
	Q4	0.557	0.961	0.843	1.097
	Q5, Ref.	–	1	–	–
35–44 years old	Q1	<0.001	0.806	0.740	0.879
	Q2	0.055	0.921	0.847	1.002
	Q3	0.561	0.975	0.896	1.062
	Q4	0.373	0.960	0.878	1.050
	Q5, Ref.	–	1	–	–
45–54 years old	Q1	<0.001	0.881	0.829	0.936
	Q2	0.186	0.962	0.908	1.019
	Q3	0.354	0.973	0.917	1.031
	Q4	0.476	0.978	0.920	1.040
	Q5, Ref.	–	1	–	–
55–64 years old	Q1	0.001	0.914	0.868	0.962
	Q2	0.125	0.962	0.917	1.011
	Q3	0.106	0.960	0.913	1.009
	Q4	0.621	0.987	0.938	1.039
	Q5, Ref.	–	1	–	–
65–74 years old	Q1	0.540	0.985	0.937	1.035
	Q2	0.962	1.001	0.955	1.050
	Q3	0.763	1.007	0.960	1.057
	Q4	0.988	1.000	0.951	1.051
	Q5, Ref.	-	1	-	-

A similar age-specific pattern was found among those aged 35–44, with the exception that residents of areas with the lowest proportion of vocationally trained individuals had a 19.4% lower mortality risk [Exp(B) = 0.806; 95% CI: 0.740–0.879; *p* < 0.001] than those living in regions with the highest concentration, and only this quintile showed a statistically significant reduction in mortality risk.

In the 45–54 age group, the association between mortality risk and the spatial concentration of vocationally trained populations remained weak. A statistically significant reduction in mortality risk was observed only in the first quintile [Exp(B) = 0.881; 95% CI: 0.829–0.936; *p* < 0.001], while no significant differences were found in the remaining quintiles.

A very similar pattern was observed among those aged 55–64 years. Again, only the first quintile showed a significantly lower mortality risk [Exp(B) = 0.914; 95% CI: 0.868–0.962; *p* = 0.001], whereas the second to fourth quintiles did not differ significantly from the reference category.

Finally, among the 65–74 age group, the association between the spatial concentration of vocational education and mortality risk weakened further. The already modest effect observed in the younger age segments had virtually disappeared in this age group [Exp(B) = 0.985; 95% CI: 0.937–1.035; *p* = 0.540].

#### 4.2.3 Upper secondary educated population

Among the upper secondary educated population aged 25–34, the relationship between educational concentration and mortality risk showed a significant inverse pattern compared with the groups with a lower level of education or vocational training. While mortality risk increased in socially disadvantaged areas among the lower educational categories, the highest mortality risk among the upper secondary educated population was found in regions with the lowest proportion of upper secondary graduates. In the least advantaged areas belonging to the first quintile, mortality risk was 47.7% higher [Exp(B) = 1.477; 95% CI: 1.301–1.676; *p* < 0.001] than in the areas with the highest share of upper secondary educated residents. In the remaining quintiles, the level of excess risk decreased progressively (Table 4).

**Table 4:** Age-specific associations between the settlement-level concentration of the upper secondary educated population and PAM risk [Exp(B), 95% CI].

Age group	Upper secondary educated population	*p*-value	Exp(B)	95% confidence interval for Exp(B)	
				Lower	Upper
25–34 years old	Q1	<0.001	1.477	1.301	1.676
	Q2	<0.001	1.380	1.241	1.536
	Q3	<0.001	1.189	1.079	1.310
	Q4	0.005	1.143	1.042	1.254
	Q5, Ref.	–	1	–	–
35–44 years old	Q1	<0.001	1.530	1.398	1.675
	Q2	<0.001	1.382	1.279	1.493
	Q3	<0.001	1.198	1.115	1.287
	Q4	0.048	1.074	1.001	1.152
	Q5, Ref.	–	1	–	–
45–54 years old	Q1	<0.001	1.382	1.296	1.473
	Q2	<0.001	1.283	1.214	1.357
	Q3	<0.001	1.131	1.072	1.192
	Q4	0.016	1.067	1.012	1.125
	Q5, Ref.	–	1	–	–
55–64 years old	Q1	<0.001	1.343	1.273	1.417
	Q2	<0.001	1.185	1.130	1.244
	Q3	<0.001	1.107	1.057	1.159
	Q4	0.165	1.033	0.987	1.082
	Q5, Ref.	–	1	–	–
65–74 years old	Q1	<0.001	1.177	1.118	1.239
	Q2	<0.001	1.128	1.077	1.182
	Q3	0.035	1.049	1.003	1.097
	Q4	0.546	1.014	0.970	1.060
	Q5, Ref.	–	1	–	–

Among the 35–44 age group, this excess mortality risk was even stronger and more pronounced: the low concentration of upper secondary educated individuals was associated with substantially higher mortality rates. In socially disadvantaged areas the magnitude of the effect was greater and the quintile order strictly followed the logic of a social gradient. Mortality risk in the first quintile was 53.0% higher than in areas with the highest educational attainment [Exp(B) = 1.530; 95% CI: 1.398–1.675; *p* < 0.001], and all other quintiles showed significant and proportionate excess risk.

Among the 45–64 age group, the relationship between educational concentration and mortality risk remained significant, though the effect gradually diminished with age. In the 65–74 age group, the impact of social background weakened further: in areas with the lowest proportion of upper secondary educated residents, the excess mortality risk remained significant [Exp(B) = 1.177; 95% CI: 1.118–1.239; *p* < 0.001], and educational differences in mortality became progressively attenuated in this age range.

#### 4.2.4 Tertiary educated population

Among the tertiary educated population aged 25–34, mortality risk did not follow a strictly linear pattern of increase. Although the trend across quintiles was not entirely progressive, it clearly indicated that mortality risk was higher in areas with a lower social concentration of tertiary educated residents—particularly in the lowest quintile. According to the model, in areas with the smallest proportion of tertiary educated individuals, mortality risk was 74.5% higher [Exp(B) = 1.745; 95% CI: 1.555–1.959; *p* < 0.001] compared with regions characterized by the highest concentration of tertiary graduates. In the remaining quintiles, mortality risk also remained significantly higher relative to the reference group (Table 5).

**Table 5:** Age-specific associations between the settlement-level concentration of the tertiary educated population and PAM risk [Exp(B), 95% CI].

Age group	Tertiary educated population	*p*-value	Exp(B)	95% confidence interval for Exp(B)	
				Lower	Upper
25–34 years old	Q1	<0.001	1.745	1.555	1.959
	Q2	<0.001	1.383	1.252	1.527
	Q3	<0.001	1.388	1.265	1.521
	Q4	<0.001	1.182	1.080	1.293
	Q5, Ref.	–	1	–	–
35–44 years old	Q1	<0.001	1.838	1.687	2.003
	Q2	<0.001	1.677	1.561	1.802
	Q3	<0.001	1.467	1.368	1.572
	Q4	<0.001	1.255	1.171	1.344
	Q5, Ref.	–	1	–	–
45–54 years old	Q1	<0.001	1.617	1.518	1.721
	Q2	<0.001	1.477	1.398	1.559
	Q3	<0.001	1.366	1.295	1.440
	Q4	<0.001	1.233	1.169	1.300
	Q5, Ref.	–	1	–	–
55–64 years old	Q1	<0.001	1.492	1.414	1.575
	Q2	<0.001	1.346	1.283	1.411
	Q3	<0.001	1.241	1.185	1.301
	Q4	<0.001	1.154	1.101	1.209
	Q5, Ref.	–	1	–	–
65–74 years old	Q1	<0.001	1.318	1.251	1.389
	Q2	<0.001	1.234	1.178	1.292
	Q3	<0.001	1.179	1.127	1.234
	Q4	<0.001	1.126	1.076	1.178
	Q5, Ref.	–	1	–	–

Among the 35–44 age group, the association between social concentration and mortality risk exhibited a distinctly structured, progressively increasing pattern across quintiles. Mortality risk was significantly higher in all quintiles compared with the reference group—the risk exceeded 80% in the first quintile [Exp(B) = 1.838; 95% CI: 1.687–2.003; *p* < 0.001] and was still 25.5% higher in the fourth quintile [Exp(B) = 1.255; 95% CI: 1.171–1.344; *p* < 0.001]. Thus, the increase in risk was statistically significant in all quintiles, following an almost linear sequence.

In the 45–54 age group, mortality patterns remained highly sensitive to the spatial manifestations of social position. The model fit was even better than in the preceding age group, although the nature of the association began to shift. In the first quintile, representing the lowest social concentration, mortality risk was 61.7% higher [Exp(B) = 1.617; 95% CI: 1.518–1.721; *p* < 0.001] than in areas characterized by the most favourable social environment.

Among the 55–64 age group, educational concentration remained significantly associated with mortality risk, although the magnitude of this effect was more moderate compared with younger age groups [Exp(B) = 1.492; 95% CI: 1.414–1.575; *p* < 0.001].

In the oldest age group (65–74 years), the regression model also confirmed the existence of an association between mortality risk and social concentration; however, the magnitude of the differences was considerably reduced relative to the younger cohorts [Exp(B) = 1.318; 95% CI: 1.251–1.389; *p* < 0.001].

The results have shown that the age-related peaks in effect intensity are strongly dependent on educational level (Table 6).

**Table 6:** Parameter estimates of age-specific models by educational level.

Education	Age group	Maximum deviation of Exp(B) from the reference (Q5)	*p*-value [Exp(B)]	Omnibus LR*χ*^2^	Omnibus Sig.
Low-educated population	25–34	0.439	<0.001	154.075	<0.001
	35–44	0.434	<0.001	363.261	<0.001
	45–54	0.514	<0.001	413.806	<0.001
	55–64	0.529	<0.001	395.548	<0.001
	65–74	0.639	<0.001	245.426	<0.001
Vocationally trained population	25–34	0.804	<0.001	22.600	<0.001
	35–44	0.806	<0.001	38.630	<0.001
	45–54	0.881	<0.001	22.360	<0.001
	55–64	0.914	<0.001	14.676	0.005
	65–74	0.985	<0.001	1.061	0.900
Upper secondary educated population	25–34	1.477	<0.001	53.584	<0.001
	35–44	1.530	<0.001	131.161	<0.001
	45–54	1.382	<0.001	147.409	<0.001
	55–64	1.343	<0.001	154.712	<0.001
	65–74	1.177	<0.001	62.695	<0.001
Tertiary educated population	25–34	1.745	<0.001	111.532	<0.001
	35–44	1.838	<0.001	296.571	<0.001
	45–54	1.617	<0.001	299.754	<0.001
	55–64	1.492	<0.001	262.825	<0.001
	65–74	1.318	<0.001	131.240	<0.001

The coloured cells highlight the age group(s) in which the educational effect reached its maximum within each educational level.

Among the low-educated population, the peak of the educational effect appeared in middle adulthood (45–64 years). Based on both the Exp(B) values and the Omnibus LR*χ*^2^ test (LR*χ*^2^ = 413.806 for ages 45–54 and LR*χ*^2^ = 395.548 for ages 55–64), it was not entirely clear whether the association was strongest in the 45–54 or the 55–64 age group; however, in both cases, the models demonstrated a remarkably good fit.

Among the vocationally educated population, the strongest effect was observed in the 35–44 age group.

Similarly, among the upper secondary educated population, the 35–44 age range also showed the greatest effect, with statistically significant differences across all quintiles and a clearly defined risk gradient.

In the tertiary educated population, the strongest mortality gradient linked to educational concentration was observed between the ages of 35 and 54.

## 5 Discussion

The most striking finding of this study is that educational inequalities are strongly embedded in the age-specific patterns of preventable avoidable mortality in Hungary, reaching their greatest intensity during middle adulthood. This finding is consistent with a recent European study (22), which found that, across successive birth cohorts born throughout the twentieth century, mortality declined significantly among highly educated populations, whereas it stagnated or even increased among populations with low educational attainment in several countries, indicating that educational inequalities continue to pose a major public health challenge across Europe. This evidence is particularly relevant for Central and Eastern Europe, where despite an overall improvement in the health status of the population, mortality inequalities remain pronounced. The direction of the association between educational level and mortality observed in our study is highly consistent with these findings, indicating that the mortality patterns associated with educational inequalities in Hungary reflect a broader European phenomenon.

Consistent with this European background, the findings of the present study provide further evidence that educational inequalities remain strongly embedded in mortality patterns in Hungary. Our results confirm that the risk of PAM at settlement-level is closely associated with the spatial distribution of educational attainment, particularly through the pronounced social gradient observed alongside concentrations of the low-educated population. Furthermore, we have shown that the effect of education varies by age group manifesting with the greatest intensity in middle adulthood and gradually diminishing in older age groups.

Mortality risk is already strongly determined by fundamental demographic characteristics: the gender gap is significant to the disadvantage of males, while the settlement structure outlines a subtle but consistent gradient in the risk of PAM. The mortality rate for males substantially exceeds that for females, and more urbanised environments are associated with a significant reduction in mortality risk, even after accounting for population size and other structural factors.

These findings suggest that a higher level of urbanisation is associated with a substantially lower mortality risk. Our results are consistent with previous evidence showing that the lower mortality risk observed in capital cities and larger urban areas can be explained by better access to healthcare, stronger health infrastructure, and higher average levels of education and living standards. This pattern aligns well with one of the fundamental principles of social epidemiology, namely that social position and spatial location substantially determine population health and mortality risk (33). This spatial persistence of health outcomes is further evident in recent empirical evidence showing that cohesive mortality clusters transcend national borders and remain apparent despite differing national healthcare structures (34).

Although regional disparities undoubtedly pose a serious public health challenge, the underlying social determinants—particularly inequalities in educational attainment—are essential to a deeper understanding of these issues. Overall, based on our data, the quintile-based measure of territorial educational concentration was the most predictive for the low-educated population, while at higher educational levels the concentration effect appeared in different forms and with decreasing intensity—except for those with a tertiary education, where the trend remained pronounced but followed a distinct pattern. Among the vocationally educated group, social concentration showed low predictive value, which may reflect their intermediate and heterogeneous social position. The relationship between educational concentration and mortality risk differed both in magnitude and structure across educational strata. Among the low-educated population, mortality differences along the quintiles were consistent, pronounced, and gradual—this group most clearly reflected the predictive imprint of health risks arising from concentrated social disadvantage. In contrast, among those with vocational education, territorial concentration had only weak predictive power, suggesting that this group represents a structurally intermediate and socially divergent position, lacking a unified risk profile.

In the group of upper secondary educated individuals, the direction of risk is reversed: it is not the areas with higher but rather those with lower proportions of upper secondary educated residents that carry greater mortality risk—that is, social deprivation in this context can be captured as a form of absence. The pattern among the tertiary educated population is more distinctive and complex: although a concentration effect is also observable, its intensity is strong but not linear, it appears to be linked to critical mass effects or contextual thresholds. This suggests that tertiary education functions not only as a protective factor but also as an indicator of the social reconfiguration of positions, relating to territorial inequalities through a different underlying logic to that of lower educational levels.

The comparison of age-specific models by educational level reveals a clear pattern: at every level of education, the association between PAM and educational attainment weakens substantially—or nearly disappears—among the oldest age group (65–74 years). This phenomenon can be explained by several epidemiological and demographic mechanisms. Firstly, the processes of health-related selection in old age—whereby individuals in the poorest health and most vulnerable social strata are more likely to have already exited the population—naturally reduce the observable strength of the social gradient. Secondly, at older ages, biological risk factors increasingly dominate, thereby diminishing the relative weight of differences arising from educational attainment (35). These results indicate that the effect of education on health status is not constant but varies across the life course, aligning with the theories of cumulative disadvantage (36) and cumulative social effects (37).

In the present study, we sought to identify the age period during which the association between educational attainment and PAM risk reaches its greatest intensity. It is important to emphasize that the observed “peak effect” or the strongest association within a given age group, does not primarily reflect biological deterioration in health status, but rather it reflects the period of life when the impact of social inequalities becomes most pronounced. This probably does not mean that the overall risk of mortality suddenly increases at this age, but that the differences arising from social position manifest most sharply in health outcomes during this stage of life.

Therefore, this age interval does not represent the peak of absolute mortality but rather the period in which the predictive power of socioeconomic factors is at its maximum. Structural differences—such as educational attainment—are most strongly associated with differences in life expectancy during these years.

Thus,

- *Among the least educated*, the association between educational level and mortality is strongest in the middle-aged population (45–64 years). This is consistent with the theory of cumulative disadvantage, which posits that disadvantages stemming from low educational attainment accumulate over time and result in severe health consequences by midlife, and are evident in the middle-aged population (36).
- *Among vocationally trained individuals*, the strongest relationship appears in the 35–44 age group, suggesting that education-related disparities in this social stratum become embedded early in the life course, producing marked health inequalities by early middle age. However, these differences do not deepen further in later life, possibly reflecting the moderating role of social mobility or life-course deviations.
- *Among the upper secondary educated*, the peak effect observed at ages 35–44 suggests that the social advantages associated with this educational level are most strongly reflected in PAM inequalities during mid-adulthood. However, the observed gradient remains less pronounced than that found among the tertiary educated.
- *Among the tertiary educated*, the peak effect between ages 35 and 54 indicates that this stratum’s structural advantages translate most forcefully into health differentials during this stage of life. In older age groups, however, these advantages appear to level off, or are increasingly overshadowed by the relative weight of biological factors.

Our findings raise the question of why the strongest age-specific association between educational attainment and PAM risk emerges in the same age group (45–54 years) at both ends of the social hierarchy among both the least and the most educated alike. This life stage represents a critical intersection of life-course and social processes, where cumulative advantages and disadvantages exert their strongest influence on mortality risk. It is therefore not coincidental that the relationship between education and mortality peaks precisely at these two extremes: among the least educated, who face the highest risks, and among those with a tertiary education, who experience the strongest protective effects (35, 38).

Numerous social studies have confirmed that mid-adulthood (35–37) represents a turning point in the life course: biologically, the risk of chronic diseases begins to increase, yet this remains an active stage of life during which the advantages and disadvantages stemming from social status exert their full impact. Age-specific analyses show that the protective effect of education is substantial across all age groups but is strongest in younger adulthood. In later life, genetic factors, lifestyle, nutrition, and other socioeconomic determinants play a larger role; however, the impact of educational inequalities persists throughout the entire life course (18). Consistent with this perspective, the strongest educational gradients in the present study were observed among individuals aged 45–54.

Moreover, the well-documented “survivor effect” helps to explain why the impact of the social gradient is not linear across age groups: it tends to be strongest in midlife and fades in older age (39). This pattern may result from the cumulative effects of social position—such as unhealthy lifestyles or chronic psychosocial stress—which manifest most clearly by midlife, before the onset of the “selective quieting” of later life. In old age, differential mortality has already removed the most vulnerable individuals, leaving behind a more homogeneous and resilient population of survivors.

Although the present study is ecological in nature and does not permit individual-level inference, the observed associations are consistent with a substantial body of evidence linking educational attainment to inequalities in mortality and health outcomes. Several theoretical frameworks may help explain these patterns.

The primary objective of this analysis was to provide a theoretical foundation for understanding the relationship between PAM and educational attainment. According to the fundamental cause theory (40), educational attainment has been proposed as a structural mechanism through which social advantages may be reproduced across the life course. Individuals with higher educational attainment have better access to information, preventive opportunities, and are more likely to benefit from emerging technologies such as screening programmes or lifestyle interventions. The theory identifies a set of fundamental social resources (including knowledge, prestige, financial capital, and social connections) through which individuals can prevent or mitigate health risks. A key argument of this study is that even as specific methods of prevention, diagnostic technologies, or treatment modalities evolve over time, inequalities rooted in social status persist in new forms. This persistence arises because groups with greater access to resources are systematically better positioned to take advantage of new opportunities for disease prevention and health protection. In the context of PAM, this mechanism is particularly critical, as access to preventive measures remains strongly stratified by socioeconomic position (40).

Another relevant theoretical framework for this study is the concept of health literacy, which describes how education enables individuals to understand and apply health-related information (41). More educated groups are better able to recognize preventable risks, communicate effectively within the health system, and use preventive services more frequently. Rydland and colleagues differentiated diseases by their level of preventability and found that educational disparities were substantially larger for conditions that are highly preventable. This finding supports the argument that health literacy and the cognitive resources required to process and interpret information, both of which are closely linked to educational attainment, play a decisive role in determining who is able to benefit from preventive opportunities. For diseases with low preventability, these educational differences largely disappear or are considerably smaller, reinforcing the interpretation that health literacy is not merely an individual skill but a structurally determined advantage.

Although the negative binomial regression model with a log-link function effectively accounts for the overdispersion observed in mortality data, it also entails several methodological limitations. The use of settlement-level aggregates does not allow for the inclusion of individual-level determinants, such as lifestyle, income, or social mobility, nor does it explicitly capture spatial autocorrelation between adjacent areas (42). Consequently, the findings describe ecological associations between educational concentration and mortality risk and should not be interpreted as direct individual-level relationships. Furthermore, the assumed linearity between predictors and the log-transformed outcome variable, as well as the heterogeneity within quintile-based groupings, may influence the unbiasedness and generalizability of the results.

The robustness of the observed associations was further supported by the sensitivity analysis. The age-specific models were re-estimated after adjustment for sex and settlement type, resulting in only negligible changes in the estimated incidence rate ratios. For example, in the 25–34-year age group, the incidence rate ratio for the lowest quintile of low-educated population concentration changed from 0.439 to 0.438 after adjustment. In the same age group, the corresponding estimate for the lowest quintile of tertiary educational concentration changed from 1.745 to 1.744. Similar negligible differences were observed across all educational categories and age groups. Importantly, the direction, magnitude, and statistical significance of the educational gradients remained unchanged, supporting the robustness of the reported findings.

Despite these methodological constraints, the model provides a robust depiction of the principal social gradients and reliably identifies the most salient concentration effects in the spatial distribution of avoidable mortality.

The univariate approach adopted in the present study provides a clear depiction of the independent effect of educational attainment; however, a deeper understanding of social inequalities requires the application of interaction models. The previously conducted two-way and three-way interaction analyses—particularly those examining the combined effects of education and sex, as well as education and settlement type—revealed additional, nuanced associations. The models exploring the three-way interaction between educational attainment, sex, and type of settlement are of particular importance, as they shed further light on the structural nature of social inequalities. These more complex models will be presented in detail in a forthcoming study, where the full interpretation of the interaction effects will also be provided.

## 6 Recommendation

### 6.1 Recommendation

The findings of the present study demonstrate that the risk of PAM in Hungary continues to be shaped by substantial and persistent educational inequalities. The pronounced territorial, educational, and age-related disparities identified in our analyses suggest that reducing preventable mortality requires interventions that extend beyond the healthcare sector alone. Addressing these inequalities calls for coordinated action across education, public health, social policy, and regional development.

#### 6.1.1 Strengthening educational attainment and lifelong learning

Educational attainment emerged as one of the most critical structural determinants of mortality risk. Consequently, policies aimed at reducing preventable mortality should prioritize educational equity throughout the life course.

Particular attention must be given to preventing early school leaving and reducing educational dropout, especially among disadvantaged populations. Early warning systems, mentoring programmes, and targeted support for students living in socioeconomically deprived areas can contribute to increasing educational attainment and, indirectly, improving long-term health outcomes.

In parallel, the adult education system should be strengthened through flexible learning opportunities that support both the social and educational reintegration of individuals with low educational attainment and the continuous adaptation of the workforce to changing labour market conditions. Expanding access to lifelong learning may help mitigate long-term social exclusion, unemployment, and the associated health disadvantages.

#### 6.1.2 Reducing inequalities in access to primary healthcare and public health services

The pronounced geographical differences observed in this study indicate that improving access to preventive services remains a key public health priority.

Mitigating the fragmentation of primary healthcare, particularly in underserved and disadvantaged regions, must be a central objective of health policy. Strengthening primary care networks and supporting multidisciplinary practice communities can significantly improve access to prevention and early intervention in peripheral areas.

In addition, efforts to reduce inequalities in health literacy should be intensified. Community-based health promotion programmes specifically tailored to populations with lower educational attainment can improve the uptake of preventive services and facilitate healthier behavioural choices. Rather than relying solely on universal health communication strategies, interventions must be adapted to local social and cultural contexts.

#### 6.1.3 Prioritising prevention in middle adulthood

A particularly important finding of the present study is that the association between educational attainment and PAM risk reaches its greatest intensity during middle adulthood, especially between the ages of 45 and 54 years. This suggests that middle adulthood represents a critical window for intervention, during which the health disadvantages stemming from social inequalities can still be effectively mitigated.

A specific emphasis must be placed on increasing participation rates in organized public health screening programmes, as Hungary’s attendance rates for several key screenings fall significantly short of the target values formulated in European and WHO guidelines.

Improving the reach of screening programmes, implementing targeted population outreach, strengthening workplace health promotion, and enhancing the early detection and management of chronic diseases can collectively contribute to reducing preventable mortality in this highly vulnerable age group.

#### 6.1.4 Implementing territorially targeted public health strategies

The observed concentration of mortality risk in areas characterized by lower educational attainment highlights the need for place-based (territorial) public health interventions. Instead of applying identical strategies across all regions, health policies must incorporate territorial targeting and allocate resources proportionately to local health needs. Integrating educational, social, and health indicators into regional planning frameworks can improve the identification of high-risk communities and enhance the effectiveness of preventive interventions.

## 7 Conclusion

Taken together, our findings suggest that reducing preventable mortality cannot be achieved through healthcare interventions alone. Educational inequalities, health literacy, and territorial deprivation constitute interconnected determinants of health that require coordinated policy responses. Investments in education, targeted prevention, and the reduction of territorial inequalities are highly likely to yield substantial long-term benefits for population health and contribute to narrowing the avoidable mortality gap in Hungary.

## Data Availability

The datasets presented in this article are not readily available because the data presented in this study are available on request from the corresponding author. The data are not publicly available due to privacy or ethical restrictions. Requests to access the datasets should be directed to Csilla Nagy, nagy.csilla@semmelweis.hu.
